# Career perspective: John B West

**DOI:** 10.1186/2046-7648-1-11

**Published:** 2012-11-07

**Authors:** John B West

**Affiliations:** 1 Department of Medicine 0623A, University of California San Diego, 9500 Gilman Drive, La Jolla, CA, 92093-0623, USA

**Keywords:** Extreme hypoxia, Hyperventilation, Maximal oxygen uptake, Distribution of blood flow, Pulmonary gas exchange, Microgravity

## Abstract

I have been fortunate to work in two areas of extreme physiology and medicine: very high altitude and the microgravity of spaceflight. My introduction to high altitude medicine was as a member of Sir Edmund Hillary's Silver Hut Expedition in 1960–1961 when a small group of physiologists spent the winter and spring at an altitude of 5,800 m just south of Mt. Everest. The physiological objective was to obtain a better understanding of the acclimatization process of lowlanders during exposure to a very high altitude for several months. As far as we knew, no one had ever spent so long at such a high altitude before. The success of this expedition prompted me to organize the 1981 American Medical Research Expedition to Everest where the scientific objective was to determine the physiological changes that allow humans to survive in the extreme hypoxia of the highest point on earth. There is good evidence that this altitude is very near the limit of human tolerance to oxygen deprivation. Much novel information was obtained including an extraordinary degree of hyperventilation which reduced the alveolar partial pressure of carbon dioxide (*P*co_2_) to about 8 mmHg (1.1 kPa) on the summit, and this in turn allowed the alveolar partial pressure of oxygen, *P*O_2_, to be maintained at a viable level of about 35 mmHg (4.7 kPa). The low *P*co_2_ caused a severe degree of respiratory alkalosis with an arterial pH exceeding 7.7. These were the first physiological measurements to be made on the Everest summit, and essentially, none has been made since. The second extreme environment is microgravity. We carried out an extensive series of measurements on astronauts in the orbiting laboratory known as SpaceLab in the 1990s. Many aspects of pulmonary function are affected by gravity, so it was not surprising that many changes were found. However, overall gas exchange remained efficient. Some of the findings such as an anomalous behavior of inhaled helium and sulfur hexafluoride have still not been explained. Measurements made after astronauts were exposed to 6 months of microgravity in the International Space Station indicate that the function of the lung returns to its preexposure state within a few days.

## Extreme altitude

My introduction to high altitude occurred in 1960 when I learned that Sir Edmund Hillary was planning a physiological expedition to the Himalayas. I applied to the scientific leader Dr. Griffith Pugh and was accepted in spite of the fact that I had previously never done any climbing. The Silver Hut Expedition as it was called was unique in that a small group of physiologists spent several months during the winter and spring of 1960–1961 at an altitude of 5,800 m (19,000 ft), about 16 km south of Mt. Everest. There, we carried out an extensive physiological program on acclimatization in a sophisticated, well-insulated wooden building that was painted silver. As far as we were aware, nobody had lived for such a long period at such a high altitude before. Subsequently, measurements were extended up to an altitude of 7,440 m (24,400 ft) on Mt. Makalu, which has an altitude of 8,481 m. These included the highest measurements of maximal oxygen uptake that have been reported to date [1]. The physiological program was very productive with many articles in top-level journals [2].

The primary purpose of the physiological program was to obtain a better understanding of the acclimatization process of lowlanders while they were living continuously at a very high altitude. The main areas of study were the cardiorespiratory responses to exercise under these conditions of extreme hypoxia, but measurements of blood, renal, and neuropsychometric function were made as well [3]. However, in the event, there was an unrelenting rapid loss of body weight, and the conclusion was that we would not have been able to remain at that altitude indefinitely.

**Figure 1:**
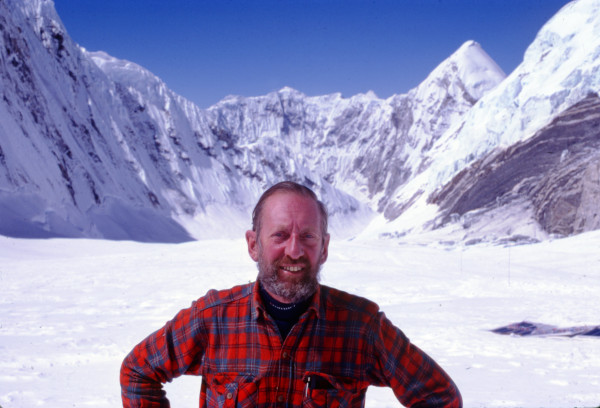
**The author near Camp 2, altitude 6,300 m, during the American Medical Research Expedition to Everest.** Everest is on the right, and Nuptse is on the left; the peak of Pumori can be seen in the distance.

**Figure 2:**
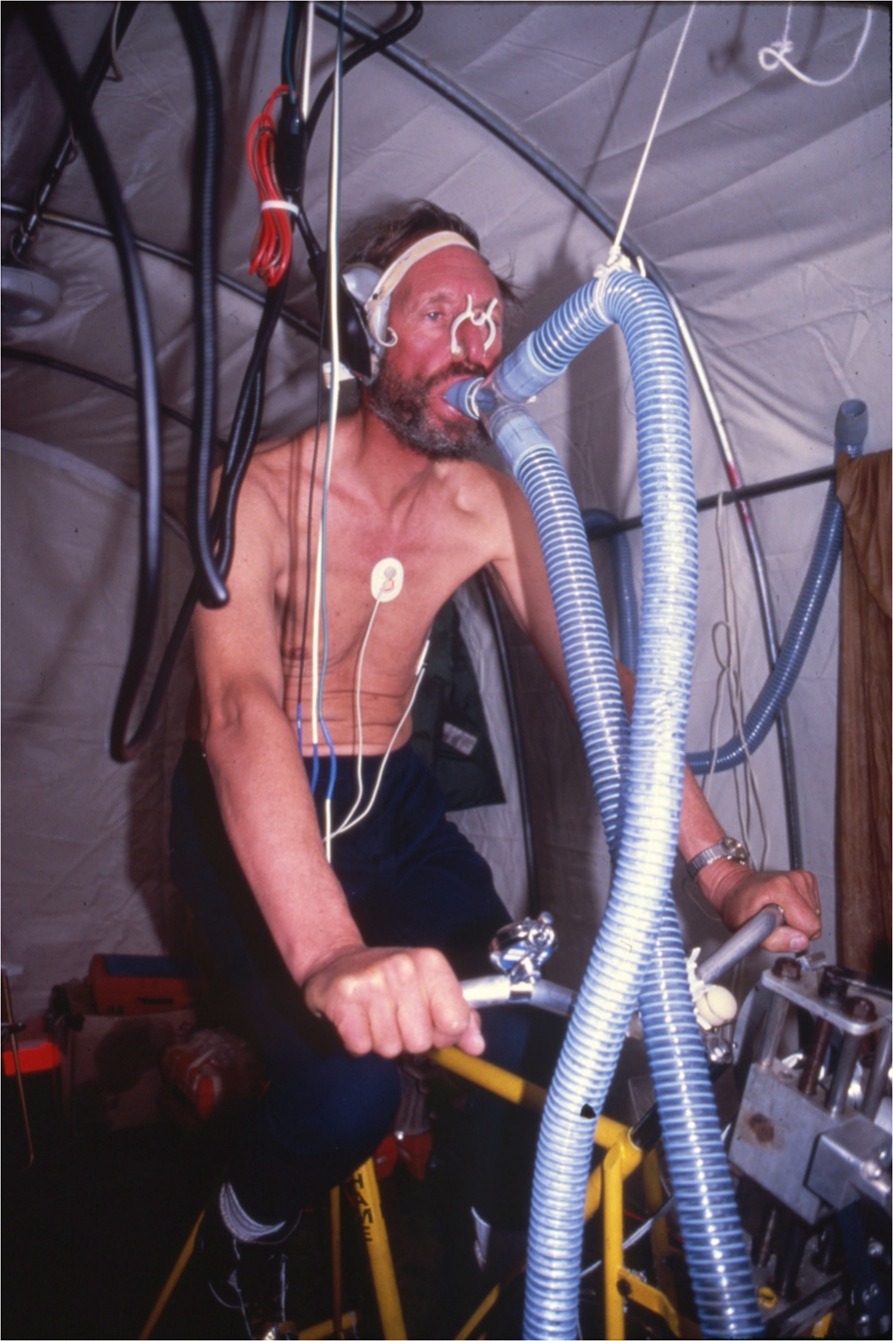
The author as a subject in measurements of exercise physiology in the Camp 2 laboratory during the American Medical Research Expedition to Everest.

The success of this expedition prompted me to wonder whether it might be possible to obtain physiological measurements at the highest point on earth. There was abundant evidence that at this altitude, humans are very close to the limit of oxygen deprivation, and so, it was a fascinating physiological problem to determine how the body responds. The opportunity occurred during the 1981 American Medical Research Expedition to Everest (Figures 1 and 2). This time, the scientific objective was to better understand the physiological changes that allow lowlanders to survive when they are exposed to extreme hypoxia of the highest point on the planet [4]. We were a fortunate expedition; in spite of a number of close calls, five people reached the summit, and a number of important measurements were made there. It might be added that previous to this, nobody had attempted to make physiological measurements at such great altitudes, and indeed, essentially, no measurements have been made on the summit since.

One of the summiteers, Dr. Christopher Pizzo, made the first direct measurement of barometric pressure on the summit at 253 mmHg (33.7 kPa), which was a little higher than what had been predicted [5]. Pizzo also collected alveolar gas samples on the summit using specially designed equipment, and these were brought back to The University of California, San Diego for analysis. They showed that the alveolar partial pressure of carbon dioxide (*P*co_2_) fell to the extraordinarily low value of about 8 mmHg (1.1 kPa), while the alveolar partial pressure of oxygen (*P*O_2_) was in the range of 35–37 mmHg (4.6–4.9 kPa) [6]. Additional alveolar gas samples were collected from five subjects at an altitude of 8,050 m. Analysis of all these data combined with measurements made at lower altitudes by previous expeditions showed that while both the *P*O_2_ and *P*co_2_ declined with increasing altitude, the successful climber was able to maintain an alveolar *P*O_2_ of about 35 mmHg (4.7 kPa) above an altitude of about 7,000 m by an enormous increase in alveolar ventilation. In this way, he was able to defend the alveolar *P*O_2_ at a viable level. We were not able to take arterial blood samples on the summit, but calculations based on the gas and blood data that we had collected indicated an arterial *P*O_2_ of about 30 mmHg (4.0 kPa). This agreed well with measurements made later during simulated ascents of Everest in low-pressure chambers [7,8] and some arterial blood samples from the Caudwell Xtreme Everest Expedition collected at an altitude of 8,400 m [9].

Another interesting finding was an extreme degree of respiratory alkalosis on the summit. Base excess measurements were made on the venous blood of two summit climbers the morning after their climb, and when these were combined with the alveolar *P*co_2_ values, the arterial pH was calculated by the Henderson-Hasselbalch equation to be between 7.7 and 7.8 [10]. A feature of this alkalosis is that it increases the oxygen affinity of hemoglobin, thus assisting the oxygen uptake in the pulmonary capillary. An increased oxygen affinity of hemoglobin is seen in many animals exposed to hypoxic environments, and it is fascinating to note that the successful climber reaches the same solution by a strategy unique to the extreme altitude environment, which is an extraordinary degree of hyperventilation.

## Microgravity of spaceflight

One of my earliest scientific projects was to study some of the effects of gravity on pulmonary function. In the late 1950s, we were lucky enough to have access to short-lived radioactive oxygen-15 (half-life, 2 min) from the newly installed Medical Research Cyclotron at Hammersmith Hospital in London. By inhaling this, we were able to show for the first time the striking inequality of blood flow down the upright human lung [11]. These and other measurements opened up a whole new field of the effects of gravity on pulmonary ventilation, regional lung expansion, intrapleural pressure, and mechanical stresses. As a consequence, I decided to take a year of sabbatical leave in 1967–1968 at the National Aeronautics and Space Administration (NASA) Ames Research Center in California, where the effects of weightlessness on the astronauts were being discussed. While there, I submitted a proposal to NASA to measure pulmonary function in weightlessness, although this was before Neil Armstrong landed on the moon, and it was not at all clear whether these studies would ever be possible. The application was funded in 1969 when I moved to the new Medical School at the University of California, San Diego, and happily, funding continued for over 30 years.

Initially measurements were made in high-performance aircraft flying in a parabolic profile pattern to give periods of weightlessness of up to about 25 s [12]. However, in the 1990s, we eventually were able to make a very extensive series of studies of pulmonary function on astronauts in low earth orbit. These measurements were made in SpaceLab, a sophisticated laboratory that was carried up in the bay of the Shuttle [13]. It provided a ‘shirtsleeves’ environment in which the astronauts were breathing air at a normal barometric pressure and oxygen concentration, and the only difference was weightlessness, or as NASA prefers to call it, microgravity.

As expected, we found a number of changes in pulmonary function under these conditions [14]. The distribution of blood flow and ventilation in the lung became more uniform, although some inequality remained. This was hardly surprising because the lung has a very complicated system of blood vessels and airways, and it is difficult to believe that all the gas-exchanging units could have exactly the same ventilation and blood flow. An interesting finding was a substantial increase in the pulmonary diffusing capacity for carbon monoxide. This was caused by both an increase in the volume of blood in the pulmonary capillaries and an increase in the membrane diffusing capacity. Both of these changes could be explained by the fact that some blood redistributes from dependent regions of the body to the chest in microgravity. Under normal conditions, blood pools in the lower regions, but this is abolished in microgravity. This headward shift of blood increases the interstitial fluid pressure in the upper part of the body and is responsible for the periorbital edema sometimes seen in crew members during spaceflight.

Lung volumes were altered. Functional residual capacity was between the values seen in the upright and supine postures in normal gravity (1G). The reason is that in the absence of gravity, the diaphragm is neither pulled down by the weight of the abdominal contents in the upright position or forced headward by the abdominal pressure in the supine position. An unexpected finding was a reduction in residual volume. This is uncommon in 1G and is possibly explained by the fact that all the alveoli have the same volume in microgravity and therefore can attain a small size before any small airway closure occurs. A big surprise was an anomalous behavior of inhaled helium and sulfur hexafluoride, two gases of very different molecular weights. The difference in behavior was presumably related to the very different diffusion rates of the two gases, but since diffusion is a mass-related (not weight-related) phenomenon, it is difficult to understand why it is affected by microgravity. The explanation of this anomalous behavior is still not clear but may have to do with changes in the geometry of the small airways in microgravity, possibly caused by subclinical interstitial pulmonary edema.

Although several aspects of pulmonary function were altered in microgravity, overall gas exchange remained efficient. Of course, this is something that NASA wanted to hear because the implication is that lung function is not likely to be a limiting factor during long-term spaceflight. Recently, measurements have also been made on crew members who have spent up to 6 months in the microgravity of the International Space Station. These measurements showed that a day or two after return to normal gravity, pulmonary function returned to its preexposure state [15]. This is in contrast with other body systems such as the musculoskeletal system where muscle wasting remains for some time after its return to normal G, and decalcification of the bone is very slow to return towards normal.

Of course, it was a special privilege to make the first physiological measurements on the highest point on earth and to carry out the first measurements of pulmonary function in the unexplored environment of microgravity. I hope that younger scientists that follow have some of the same opportunities.

## Abbreviations

*P*co_2_: Partial pressure of carbon dioxide; *P*O_2_: Partial pressure of oxygen.

## Competing interests

The author declares that he has no competing interests.

## Authors’ information

JBW obtained his medical degree in Adelaide, Australia, and after a year of residency, he moved to London where he spent the next 15 years at the Postgraduate Medical School, Hammersmith Hospital. With others, he described the uneven distribution of blood flow in the upright human lung and went on to explore other effects of gravity on respiration. In 1960, he was invited by Sir Edmund Hillary to take part in the Silver Hut expedition described in this article. He continued his interest in high altitude medicine by leading the 1981 American Medical Research Expedition to Everest which is also described here. His interests in the effects of gravity on the lung led him to work with NASA, and he was the principal investigator on a series of experiments on orbiting astronauts to describe the effects of weightlessness on pulmonary function. Some of these are described in the present article. He is a dedicated teacher, and his small books, *Respiratory Physiology: The Essentials* and *Pulmonary Pathophysiology: The Essentials*, have been translated into several languages and are used throughout the world. His video lectures on YouTube (http://meded.ucsd.edu/ifp/jwest/) are also used extensively. In 1969, he was recruited to the new Medical School at the University of California, San Diego where he has remained as professor of Medicine and Physiology.
